# Adverse Social Determinants of Health in Children with Newly Diagnosed Type 1 Diabetes: A Potential Role for Community Health Workers

**DOI:** 10.1155/2024/8810609

**Published:** 2024-01-23

**Authors:** Charlene W. Lai, Meghan Craven, Jennifer A. Hershey, Terri H. Lipman, Colin P. Hawkes

**Affiliations:** ^1^Division of Pediatric Endocrinology, Oregon Health and Sciences University, 700 SW Campus Drive, Portland, OR 97239, USA; ^2^Division of Pediatric Diabetes and Endocrinology, Baylor College of Medicine, 6701 Fannin Street Suite 1020, Houston, TX 77030, USA; ^3^Children's Hospital of Philadelphia, 3400 Civic Center Boulevard, Philadelphia, PA 19104, USA; ^4^School of Nursing, University of Pennsylvania, 418 Curie Boulevard, Philadelphia, PA 19104, USA; ^5^Perelman School of Medicine, University of Pennsylvania, 418 Curie Boulevard, Philadelphia, PA 19104, USA; ^6^Department of Paediatrics and Child Health, University College of Cork, Cork, Ireland; ^7^INFANT Research Centre, University College Cork, Cork, Ireland

## Abstract

**Objective:**

There are significant socioeconomic and racial disparities in glycemic control among children with type 1 diabetes (T1D). Community health workers (CHWs) have been shown to improve outcomes in marginalized, high-risk populations. The purpose of this qualitative study was to describe the prevalence and the impact of adverse social determinants of health (SDOH) on diabetes care soon after a diagnosis of pediatric T1D, and investigate the potential supportive role of a CHW. *Research Design and Methods*. Caregivers of youth <17-year old, with new onset T1D, and government insurance at the time of diagnosis were enrolled. Baseline demographic and SDOH questionnaires were administered at the time of enrollment. Semistructured interviews were performed at 3 months after diagnosis to explore the effect of SDOH on diabetes care and the impact of a CHW.

**Results:**

Seventeen caregivers were enrolled, 10 were randomly assigned to a CHW. Two-thirds of caregivers identified at least one SDOH need at enrollment; 35% of caregivers identified two SDOH needs. Interviews revealed that the two major themes identified as barriers to diabetes care were caregivers' employment and financial issues. Social support was identified as a facilitator. The transition from hospital to home after the diagnosis of T1D was improved for families working with a CHW, and the CHW was identified as a strong source of support.

**Conclusions:**

There is a high prevalence of adverse SDOH in families from lower socioeconomic status at the time of diagnosis of pediatric T1D. These SDOH have a significant impact on families' abilities to care for their children. Preliminary data suggest that CHWs can be a facilitator to the diabetes care. This trial is registered with NCT04238949.

## 1. Introduction

Despite significant advances in the care of children with type 1 diabetes (T1D), there are persistent socioeconomic and racial disparities in outcomes that include glycemic control, prevalence of severe hypoglycemia, quality of life, and healthcare utilization [1–4]. Consequently, a disproportionate number of children from vulnerable, underserved populations experience higher rates of diabetic ketoacidosis and long-term complications [5]. These disparities in healthcare outcomes emerge almost immediately following diagnosis of T1D [6].

International best practice recommends the delivery of a structured education program for all children following onset of T1D [7]. We have previously shown that intensifying education support during the first year after diagnosis can improve glycemic control, but only in children from higher socioeconomic status (SES). The absence of a demonstrable effect among children from lower SES families highlights the confounding and often under-appreciated role of social determinants of health (SDOH) on diabetes outcomes [2, 3, 8–11].

Community health workers (CHWs) are trained members of the community who provide social support and advocacy to under-resourced patients. CHWs meet families outside the hospital and partner with them to help access local resources, interact with the medical team, and address SDOH challenges [12–14]. Increasingly, the addition of CHWs to the healthcare team is showing a positive effect on outcomes in patients from lower SES. The addition of a CHW has demonstrated improved disease management and reduced healthcare utilization in children with asthma, children with sickle cell disease, and adults with diabetes and other chronic diseases [12, 15–18]. Various roles of CHWs in healthcare have been described, and the implementation of this intervention varies among healthcare settings.

## 2. Purpose

The aim of this qualitative study was to describe the impact of adverse SDOH on diabetes care in children from lower SES in the first few months from diagnosis of T1D. We also sought to describe the role of CHWs in addressing SDOH in children with newly diagnosed T1D.

## 3. Methods

This qualitative study was comprised of interviews conducted with caregivers of children from low-income families 3 months from diagnosis of T1D. This study was performed as part of a longitudinal randomized controlled trial exploring the addition of CHWs to the care teams of children from low-income families with newly diagnosed T1D (NCT04238949). The study was approved by the Children's Hospital of Philadelphia Institutional Review Board.

### 3.1. Study Setting and Participants

Enrollment occurred in person and by telephone at a single tertiary care academic pediatric hospital between November 2019 and July 2021. Eligible participants were caregivers of the children who (1) were <17-year old, (2) were within 31 days of a clinical diagnosis of T1D, (3) had Medicaid or Children's Health Insurance Plan at the time of diagnosis, (4) had at least one diabetes autoantibody positive, and (5) were English speaking with an English-speaking caregiver. The source of primary medical insurance was used as a surrogate for SES, with government insurance (Medicaid or Children's Health Insurance Plan) representing low SES. In the United States, there is not universal access to healthcare, and most families receive medical care through private insurance. Only families with low-income qualify for government insurance. As a part of the larger study, participants were randomly assigned to one of two arms (1) standard diabetes care and (2) addition of a CHW to their diabetes team.

### 3.2. Allocation to Treatment Groups and Blinding

After informed consent and determination of eligibility, a researcher external to the study performed randomization. This was completed using a computer-generated randomization list stratified in randomized blocks according to patient sex and patient age group (age younger than 12 years, and age 12 years and older).

### 3.3. Initial Assessment

Baseline sociodemographic and Health Leads Social Determinants of Health questionnaires were completed at the time of recruitment [19].

### 3.4. Interviews

Semistructured interviews were conducted with the participants. Although these interviews were intended to occur 3 months after recruitment to the study, the timing of the initial interview extended up to 9 months in the context of the COVID-19 pandemic. A semistructured qualitative interview guide was created to explore how SDOH play a role in how parents cared for their child's diabetes (Supplementary Materials). Questions were focused on the environment in which families live, work, learn, and play [20]. The interview guide consisted of guiding questions that allowed the interviewer flexibility in tailoring follow-up and probing questions to best fit the interviewee. The interview guide was reviewed to ensure use of neutral language and included open-ended questions that would allow participants to express their own views without prompting. Interviews were performed over telephone by a member of the research team (C. L. or M. C.). Interviewers were trained through the Qualitative Research Methods Course at the University of Pennsylvania.

### 3.5. Children in the Community Health Worker Group

In addition to the standard diabetes care, 10 participants were randomly assigned to have a CHW included as part of their diabetes team. There were three CHWs. Two of the CHWs graduated from Temple University's CHW Training Program and a third CHW was previously a behavioral healthcare coordinator [21]. All CHWs were certified through the Pennsylvania Certification Board. CHWs spent time shadowing and participated in weekly clinical team meetings with the Diabetes Center providers. They were supervised by a licensed social worker. CHWs' work with these families included goal setting, goal support, and connection with the healthcare system. CHWs completed a comprehensive assessment: speaking with the patient, caregivers, school personnel, diabetes provider, and diabetes social worker to collaborate with families on identifying patient- and family-centered goals. CHWs worked with caregivers and patients to create an individualized plan for achieving each of these goals. Plans consisted of a measurable goal, caregiver confidence in achieving the goal, resources, and a step-by-step plan for goal achievement.

### 3.6. Analysis

Interviews were audio-recorded and transcribed verbatim using the Datagain Transcription Services (Secaucus, NJ, USA). Two researchers (M. N and R. N.) performed a thorough reading of transcripts to create a codebook using a modified content analysis approach that also allowed for emergent themes. This codebook was applied to all transcripts using NVivo 1.5 (QSR International, Burlington, MA, USA). A portion of the transcripts were cocoded (23%, *N* = 4) and inter-rater reliability was periodically assessed to assure agreement in codebook application. Coding outputs were then analyzed.

## 4. Results

**Figure 1:**
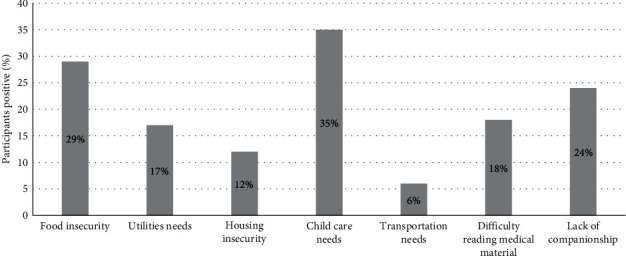
Baseline adverse social determinants of health; results of health leads SDOH questionnaire. ^*∗*^No one screened positive for concerns around cost of seeing a doctor or safety.

There were 27 caregivers enrolled in this study. Ten were withdrawn and were not included in the analysis. Reasons for withdrawal were: did not complete baseline surveys (*n* = 3), diabetes autoantibody negative (*n* = 3), transfer of care to another institution due to insurance (*n* = 1), ineligible insurance type (*n* = 1), subsequent enrollment in a drug study (*n* = 1). One participant who was randomized to the CHW arm subsequently chose to withdraw from the study. The study was designed so that randomization occurred after the baseline survey was completed. Therefore, the three participants who did not complete baseline surveys were withdrawn from the study before randomization. Of the remaining 17 caregivers, 10 had a CHW assigned to their care team. The mean patient age was 8.4 years and 67% were female; 41% were Black and 23% Hispanic. Interviews took place from 89 to 273 days following randomization (median 119 days (IQR 98, 149)). At the time of enrollment, 59% of caregivers identified as single parents, 70% had household incomes <$35,000 per year, 53% had high school degrees or less, 29% were under or unemployed, 59% rented their current residence, and 82% were receiving at least 1 type of government assistance in addition to the government health insurance (Table 1). Half of the participants were receiving Supplemental Nutrition Assistance Program (SNAP) benefits. Twenty-two percent of participants received monthly payments through the Supplemental Security Income (SSI), and 18% were receiving utility assistance through the Low Income Home Energy Assistance Program (LIHEAP). At the time of recruitment, based on the Health Leads SDOH Questionnaire, 65% of caregivers reported at least one SDOH need, and 35% of caregivers reported at least two SDOH needs with a range from 0 to 5 adverse SDOH positive. Of the nine SDOH domains assessed, the top four most commonly reported needs were child care challenges (35%), food insecurity (29%), utility insecurity (17%), and social support (24%). No one screened positive for concerns around cost of seeing a doctor or safety in the home (Figure 1).

Among the 10 caregivers who had a CHW assigned to their care team, the most common support provided by the CHW in the first 3 months of intervention included: school related diabetes issues (IEP/504 and school nurse meetings (*n* = 4)), help with housing insecurity through navigating home insurance claims, rental home listings, and rental assistance programs (*n* = 3), connecting to home energy assistance (*n* = 2), and connecting to diabetes support groups (*n* = 2).

### 4.1. Aim 1: The Impact of SDOH on Early Diabetes Care

Caregivers identified employment, finances, social support, child care, and school as SDOH affecting diabetes care (Table 2).

#### 4.1.1. Employment and Childcare

Challenges with childcare were the most commonly identified adverse SDOH identified at time of enrollment. On the Health Leads SDOH survey, 35% (6/17) of all participants identified that issues with childcare made it hard to work or study in the last 12 months. During the qualitative interviews, many participants stated that their work schedule interfered with having a preferred level of oversight of their child's diabetes care. Some participants reported that their child was attending school remotely and had little to no adult supervision of diabetes care during the day. Others were concerned that school staff and other caregivers responsible for their child while they were at work did not fully understand the demands of diabetes care and management.

Barriers to effectively caring for their child's diabetes included not being home to regularly check their child's blood glucose, monitor their diet, or attend medical appointments. Participants who were able to have a partner or other caretaker stay at home with their child, or who were home from work due to the pandemic, were relieved that their child was being cared for by someone who understood diabetes maintenance.

*“My husband was [working] full-time prior to diagnosis. Since diagnosis, he's been home. So, at this point, it's more of someone's gonna have to stay behind because somebody has to give [our child] care that's 24 hours that actually knows what they're doing. So, it's either him or me and at this point, I can make more than what he can. So, it's more of him staying home to take care of him while I still work and do what I have to do because income can shift. And it's easier for us to have someone, either me or him, who does everything, to actually do everything for him.” (1023*)

#### 4.1.2. Insurance and Finances

Many participants identified insurance concerns or financial constraints as barriers to caring for their child's diabetes and felt that uncertainty around their financial situation or employment added to their stress.

The baseline SDOH screens identified that 29% (5/17) of participants ate less in the last 12 months than they felt they should because there was not enough money for food. Seventeen percent (3/17) reported in the last 12 months that a utility company had shut off service for not paying their bills. Twelve percent (2/17) reported concerns that in the next 2 months they may not have stable housing.

In addition, interviewees identified concerns around access to their child's medication, consistent medical attention, and remembering to pay bills while balancing their other responsibilities were identified as especially difficult for some participants. Some participants felt that changing their child's diet to help them in managing their diabetes was also a significant financial impact.

#### 4.1.3. Social Support

Social support was a positive factor for most families. Many interviewees frequently cited a close social support system of family members and friends that helped them care for their child, including spouses or partners, other children, and extended family members such as grandparents, aunts, or uncles. Participants also gained valuable insight and understanding from the members of their extended social networks, including teachers, healthcare professionals, and acquaintances of all kinds who shared their experiences with diabetes or caring for someone with the disease. On the SDOH screen, while the majority of interviewees identified multiple sources of social support, 24% (4/17) of participants identified that they lacked close companionship.

Many participants also relied upon online resources including message boards, Facebook pages, nutrition tracking apps, and support groups for information and assistance in managing their child's diabetes care. While the majority of participants reported easily accessing these resources and finding the available information helpful, a minority did not access them or felt that they were not technically savvy enough to benefit from participating in the online support.

*“I reach out to his therapist [for support] and I also am friends with about five or six [social media] groups … There's parents, there's teenagers, there's even adults. And even my physical therapist is a diabetic and he was telling me it was hard when he got diagnosed with it and around Christmas time, he was around my son's age, same thing. So, yeah, [I'm] trying to get help from a lot of people.” (1021)*

#### 4.1.4. Impact on School

The experiences of participants to help their child return to regular schooling after the diabetes diagnosis were highly variable. Some participants felt fully supported by their child's school's infrastructure and were in close contact with nurses, counselors, and teachers to help their child transition smoothly back to the classroom. Others felt adrift and were even asked to keep their child home from in-person learning due to the volatility of their child's diabetes. For participants who had the flexibility to be home while their child was attending remote class due to COVID-19, they could closely monitor their health and diet. Other participants reported relying on older siblings to assist in the care of their child with diabetes during the school day while they were required at work.

*“She went back to regular [in-person] school. At first she wanted to go, but she was kind of scared, like, you know, different scenarios, like what would happen, who would help her? But definitely, because I'm the [Participant's job], I had relationships with this principal, teachers and so on. And she's been in that school since she was in preschool, so, they kind of rallied around her and, you know, helped her in any way that they can. And then she also because a lot of kids asked her like about the [monitor] and so on. She actually got together with the nurse, and they did a presentation for her class, so that they would be aware of, you know, what diabetes is.” (1019)*

*“Her school is a preschool; they don't have a nurse. So that was the only thing and then her director of the preschool will have her take [diabetes education] class in order for her to go back. And [the director] wasn't comfortable taking that class, it was too much. So, this is where I'm at.” (1013)*

### 4.2. Aim 2: The Roles Played by CHWs in Addressing SDOH

This analysis included the 10 caregivers who had a CHW assigned to their care team. The majority of participants had highly positive experiences with their CHW and felt comfortable going to them when they needed support, additional resources, or assistance in caring for their child's diabetes. Participants felt that their CHW was a key contact should they need support. These participants were pleasantly surprised at the array of services, resources, and supports their CHW could provide, from emotional support through a simple phone conversation to helping find a reliable contractor for home repairs. Other supports and resources received by participants from their CHW included assistance with communicating their child's health needs to school administrators, transportation, diabetes education, relocation and housing resources, applications for government food benefits, and information about COVID-19-related business relief, among other things.

*“They call and check on you, they help you with resources. They help you set up appointments to help you reach insurance company or doctors if they want to give you the runaround. Trust me, she's helped me a lot with that. She's actually awesome. You can also like, call her friend, because like, she's there for me, him or her to talk to or advice without judgment.” (1021)*

*“I think she just makes sure that we're on track. I know that she's available, that if I was having trouble with insurance. I know she gave me contact information for getting my roof fixed, which I just haven't had a chance to deal with school, the way that my schedule is online. And then by the time I'm done online, the last thing I want to do is look at a computer screen and talk about my roof. But, again, she's been very helpful, which is just checking in on us, just to see if our needs are being met, the basic needs and then also if we have any resources that she can help us find if we needed them. Then in that sense, it's been very helpful.” (1008)*

Two participants felt that their link with their CHW was tenuous. These participants did not feel that they had a strong relationship with the CHW and were less certain of the benefits of the relationship. Barriers to positive sentiment toward their CHW often related to inconsistent communication, different geographic locations, or not having a strong interpersonal relationship with their CHW.

For participants who were not assigned a CHW (*n* = 7), their experience navigating the health system was described as markedly more difficult. These participants felt that support from their child's care team was unavailable to them after diagnosis. Some reported feeling overwhelmed by “information overload” or having difficulty remembering all of the information they needed to keep their child healthy.

*“There's no support. I'm saying I feel like once I left the hospital, the support left. [Hospital] is the bomb when you're in the hospital, right? … I don't feel like there's enough support and then they don't give you the resources of, ‘Here, look, this is who you should call or what you should do.' I feel like I went into this kind of blindsided. A lot of stuff that I was told I didn't expect.” (1013)*

## 5. Discussion

Children from families with lower incomes and living with adverse SDOH have worse glycemic control, and this emerges early in the course of this disease [1–4, 6]. Given that early glycemic control predicts future T1D outcomes, it is especially important to identify and address disparities early [22]. In this study, we sought to better understand how adverse SDOH affect families soon after a new diagnosis of pediatric T1D, as well as the potential roles of CHWs in supporting families in the first 3 months after diagnosis. To our knowledge, this is the first study to explore the SDOH challenges experienced by families with low income at the time of newly diagnosed T1D. We have identified common themes that emerged as barriers to diabetes care at this critical time such as lack of social support, as well as negative impacts on school, childcare, and finances. Further, when a CHW was added to the care of some families in this study, they played a critical role in connecting families to additional supports and assisted with integrating the child with T1D back into school and other life areas.

While we know from other studies that adverse SDOH impact glycemic control, there are limited data on how adverse SDOH impacts the initial experiences of caring for a child with T1D and the transition from hospital to home [10, 23, 24]. Food insecurity is the most commonly described adverse SDOH in diabetes research. In our study, the Health Leads SDOH questionnaire identified that 29% of families had food insecurity in the last year. This is consistent with the SEARCH for diabetes in youth study in 2021 [25]. In addition, in our qualitative interviews, many parents expressed that buying healthier food options for their family after their child was newly diagnosed with T1D was a financial burden. Similar observations have been made in other qualitative studies. A qualitative study examining impacts of SDOH in single parents of Black children with T1D reported the economic burden of food cost a major barrier [26]. Cox et al. [23] also reported in their T1D population that food insecurity had a disproportionate impact on families after T1D diagnosis compared with before diagnosis. This argues for a more proactive approach to addressing barriers early on.

In addition, studies have shown increased feelings of isolation caring for children with T1D and, in particular, parental stress around times of transition such as starting school [27]. This may be exacerbated in populations who report a lack of social support. In our study, many participants described their work schedule as preventing them from caring for their child's diabetes as they wished. Barriers to childcare was one of the most consistent challenges described by the parents. Parents often described that they did not trust that the individuals responsible for their child's diabetes care while they were at work had enough knowledge of diabetes management. There was also a broad range of experiences regarding integration back to school. Some families described a smooth transition of trusting relationships with staff, while another parent described how, due to the lack of a school nurse, she had to go to school daily to administer insulin.

For the families that were assigned a CHW in our study, CHWs started building relationships with families, addressing social needs, and working on goal setting. They began addressing the main SDOH barriers identified by families and aided with a wide variety of services ranging from emotional support for the parents, to diabetes care coordination, assistance with housing, access to food, and troubleshooting insurance problems. The CHWs were generally well-received by participants in the program and were described as facilitators to care. Participants described them as a friend, a source of support, and a key contact and connection to the Diabetes Center. There is little published literature on patient perspectives on similar interventions in this population, but Malik et al. [28] leveraged both lay health workers and attorneys to improve diabetes outcomes in pediatric T1D and also showed high levels of participant satisfaction. Other studies examining patient perspectives of working with a CHW for adult patients with chronic diseases such as type 2 diabetes, HIV, obesity, or hypertension also reported high levels of satisfaction [18, 29, 30]. While CHWs in this study were able to address many of the common barriers such as lack of support, they did not address lack of childcare. This SDOH barrier can be difficult to address, but is integral to diabetes outcomes. CHWs could assist with this in the future by identifying appropriate caregivers and helping them connect to the Diabetes Center for comprehensive diabetes education.

While there has been an increased focus on descriptions of disparities based SDOH, few interventions have been described [31]. This study demonstrated that adverse SDOH affect diabetes care from the very beginning of diabetes care, and that CHWs are an intervention that may be able to improve SDOH in this vulnerable population.

## 6. Strengths and Limitations

This study includes racially diverse families from lower SES, a population that is under represented in research and is more likely to have worse diabetes outcomes. The mixed methods approach of questionnaires and semistructured interviews also provide a comprehensive overview and exploration of the challenges experienced. The single center nature of the study is a limitation, although it is likely that the adverse SDOH experienced in this study group is generalizable given the similar findings in other studies. Additionally, there are inherent limitations associated with semistructured interviews as a research method, such as interviewer bias and the open-ended nature of questions, which may result in incomplete information from participants. Non-English speaking families were excluded from the study to avoid the confounding effects of the use of an interpreter in a relationship-based intervention. And lastly, the four participants, who were withdrawn due to lack of participation may have had significant SDOH challenges that prohibited them form completing the study, therefore underestimating the baseline SDOH needs of the study population. This withdrawal rate is high and recruitment and retention of vulnerable families in research is often a challenge [32–34]. In addition, this study was completed mostly during the COVID-19 pandemic. The pandemic likely posed additional challenges to families and changed the CHW intervention from in person to virtual.

## 7. Conclusion

It is crucial not to overlook the impact of adverse SDOHs on the ability of families with low income to care for their children after a new diagnosis of T1D. These children have worse glycemic control and other diabetes outcomes compared to their higher income counterparts. Unfortunately, these SDOH challenges are frequently unaddressed. Our initial data suggest that CHWs can be a facilitator to diabetes care in this vulnerable group. Qualitative and quantitative data at 1 year after diagnosis will further illuminate families' experiences of caring for a child with T1D with and without the support of a CHW.

## Figures and Tables

**Table 1:** Demographics of participants enrolled, at the time of recruitment.

Number of participants (*N*)	17	

Patient age (mean)	8.4 ± 3.1	
pH <7.3 at presentation	10 (59%)	
Race/ethnicity	Black (41%) NHW (23%) Hispanic (23%) Other (12%)	
Adults in home (median)	1 (Range 1–4)	
Children in home (median)	3 (Range 1–5)	

Caregiver status		
Single parent or lone parent	10 (59%)	

Caregiver education		
Less than high school	2 (12%)	
High school degree	7 (41%)	
Higher education	8 (47%)	

Employment status of caregiver and other caregiver (if applicable)		
	Caregiver	Other caregiver
Unemployed	5 (29%)	3 (30%)
Employed (part-time or full-time)	12 (70%)	7 (70%)

Household income		
<$25,000	5 (29%)	
$25,000–$34,999	7 (41%)	
≥$35,000–$49,999	5 (29%)	

Baseline governmental benefits received		
SSI (supplemental security income)	4 (24%)	
Section 8 or HUD (housing)	1 (9%)	
LIHEAP (energy assistance)	3 (18%)	
SNAP (nutrition)	8 (47%)	
WIC (nutrition)	2 (12%)	
None	3 (18%)	

**Table 2:** Major SDOH themes identified.

Theme	Examples of themes	Community health worker impact
Work and childcare	“If I'm gonna leave her with someone who don't know how to manage diabetes, it can be dangerous. I'm not gonna be in peace, and it can be dangerous for her, so I cannot leave her with someone, like I have some neighbors, I have some friends, I have a babysitter from her baby time, and that can play and they can watch, of course, but they don't know how to deal with diabetes.” (1027)	“She keep working, she also works with [social worker], the social worker at the endocrinology, they keep sending the letters and request for the insurance company to provide me the private nurse, like the nurse that can come to my house and stay with [participant's daughter], in case I have to do some tests or something, like I have to do some.” (1027)

Insurance and finances	“I can see it going to possibly be a challenge at some point. Right now, thankfully, we're able to qualify for food stamps … but the amount that we're getting is a lot because of the COVID-19. So once that stops, I think it'll stop in September, then I'll definitely be really budgeting everything to try and figure out how to afford to eat more … and just like eating healthy is more expensive typically, and then throwing in the celiac, it is super expensive…But I foresee it in the future happening again.” (1024) “And here's the thing, I'm not comfortable by any means, no. We live paycheck to paycheck, but I have a paycheck. It's gonna sound odd, but knowing that there's this program that's available … it's a comfort, even though we haven't needed to use any of the resources that may be out there, it is a comfort knowing that they're out there for other people who may need them.” (1008)	“Yeah, she was trying to refer me to like some rental assistance. And a lot of the places that I called, it's like COVID-19 related you had to be to get the rental assistance. There was one place that did help me and they were able to help me once a year, once a year. So, they did help me towards December, so I just re-applied with them from March.” (1016) “So I mean, he's helping me right now trying to get my tax returns from last year and this year, because they held them last year, because one of my employers reported my leasing, so I haven't gotten that, no stimulus money … and my tax return is being held again now this year … I have to find a tax advocate on their end, to look into it for me. I've called them like three times, they said they're gonna call me back, they never called me back. There's a constant battle so [CHW] said he was gonna do like a conference call, or we'll do it in person (together).” (1022)

Social support	“Everybody says there's so many support groups. I felt like when I left the hospital, it got extremely hard, I know [my partner], he's been trying to help me and I'm real bad with this internet communication stuff. I'm like the worst like, even my teacher said, I sent her an email, you've got to call me because it's hard. Checking emails is the worst.” (1013)	“I feel like her role is just actually, I look at it more like … a friend, like someone who's there to confide in to basically help with resources and get us in contact with the proper help that we might need … But yeah, with [CHW's name], she's able to help us and just literally help us with our, like me how I felt, how can I take this? I had a moment where I felt like I wasn't being the mom that I should be, right. And then with her, what's the word I'm thinking of? It's like, literally right there. She gave me like that pep talk.” (1012)

Impact on school	“The school, they were understanding but not very understanding, because I have to get him up for the first month at two in the morning and he was tired and won't get up for class and they weren't very lenient, they were like he is late, if sugar was high, they will get mad, because I'm like, “Listen, he has to get up, we got to check his sugar, if it is high, I got to correct it,” they will get mad. So, it was just crazy and then when they're supposed to take the PSSAs, they actually opted [Child] out of it, because they didn't want him coming to school taking it and then his sugar like wasn't stable… I opted for him to go in person … and then his guidance counselor called me like a week later, she like me, the nurses, teachers or principals talked and we feel like it's best for [Child]'s health that he just stay home and will excuse him for the PSSAs.“ (1021)	“The only thing is that 504 Plan, we didn't get that until in March when this COVID-19 hit. It's like he didn't even need it now because they were staying home. Yeah, (the school administrators) were playing around like that. They were just giving me all these other excuses. I don't know what it really was. Then finally, [CHW's Name], reached out to them more than just what I would do it. She got on that more. I think that was a help too, that it's a serious thing.” (1002)

## Data Availability

To access data supporting conclusions of the study, please email laic@ohsu.edu.

## Supplementary Materials

Supplementary Materials Semistructured interview guide.

## References

[1] Lipman T. H., Smith J. A., Patil O., Willi S. M., Hawkes C. P. (2021). Racial disparities in treatment and outcomes of children with type 1 diabetes. *Pediatric Diabetes*.

[2] Mendoza J. A., Haaland W., D’Agostino R. B. (2018). Food insecurity is associated with high risk glycemic control and higher health care utilization among youth and young adults with type 1 diabetes. *Diabetes Research and Clinical Practice*.

[3] Addala A., Auzanneau M., Miller K. (2021). A decade of disparities in diabetes technology use and HbA_1c_ in pediatric type 1 diabetes: a transatlantic comparison.. *Diabetes Care*.

[4] Willi S. M., Miller K. M., DiMeglio L. A. (2015). Racial–ethnic disparities in management and outcomes among children with type 1 diabetes. *Pediatrics*.

[5] Bergmann K. R., Nickel A., Hall M. (2022). Association of neighborhood resources and race and ethnicity with readmissions for diabetic ketoacidosis at US children’s hospitals. *JAMA Network Open*.

[6] Tremblay E. S., Liu E., Laffel L. M. (2022). Health disparities likely emerge early in the course of type-1 diabetes in youth. *Journal of Diabetes Science and Technology*.

[7] Lindholm Olinder A., DeAbreu M., Greene S. (2022). ISPAD clinical practice consensus guidelines 2022: diabetes education in children and adolescents. *Pediatric Diabetes*.

[8] Williams D. R., Costa M. V., Odunlami A. O., Mohammed S. A. (2008). Moving upstream: how interventions that address the social determinants of health can improve health and reduce disparities. *Journal of Public Health Management and Practice*.

[9] Malik F. S., Sauder K. A., Isom S. (2022). Trends in glycemic control among youth and young adults with diabetes: the SEARCH for diabetes in youth study. *Diabetes Care*.

[10] Zuijdwijk C. S., Cuerden M., Mahmud F. H. (2013). Social determinants of health on glycemic control in pediatric type 1 diabetes. *The Journal of Pediatrics*.

[11] Mönkemöller K., Müller-Godeffroy E., Lilienthal E. (2019). The association between socio-economic status and diabetes care and outcome in children with diabetes type 1 in Germany: the DIAS study (diabetes and social disparities). *Pediatric Diabetes*.

[12] Kangovi S., Mitra N., Grande D. (2014). Patient-centered community health worker intervention to improve posthospital outcomes: a randomized clinical trial. *JAMA Internal Medicine*.

[13] Rogers H. E., Hershey J. A., Morone J. (2023). Perspectives of pediatric community health workers: roles, successes, and challenges. *Health Promotion Practice*.

[14] Lipman T. H., Smith J. A., Hawkes C. P. (2019). Community health workers and the care of children with type 1 diabetes. *Journal of Pediatric Nursing*.

[15] Shreeve K., Woods E. R., Sommer S. J. (2021). Community health workers in home visits and asthma outcomes. *Pediatrics*.

[16] Smaldone A., Findley S., Manwani D., Jia H., Green N. S. (2018). HABIT, a randomized feasibility trial to increase hydroxyurea adherence, suggests improved health-related quality of life in youths with sickle cell disease. *The Journal of Pediatrics*.

[17] Kangovi S., Mitra N., Grande D., Huo H., Smith R. A., Long J. A. (2017). Community health worker support for disadvantaged patients with multiple chronic diseases: a randomized clinical trial. *American Journal of Public Health*.

[18] Kangovi S., Mitra N., Norton L. (2018). Effect of community health worker support on clinical outcomes of low-income patients across primary care facilities: a randomized clinical trial. *JAMA Internal Medicine*.

[19] (2018). The Health Leads Screening Toolkit.

[20] Healthy People 2030 Social Determinants of Health (2023). Social Determinants of Health.

[21] Temple University Community Health Worker Lenfest Center for Community Workforce Partnerships.

[22] Mazarello Paes V., Barrett J. K., Taylor-Robinson D. C. (2019). Effect of early glycemic control on HbA1c tracking and development of vascular complications after 5 years of childhood onset type 1 diabetes: systematic review and meta-analysis. *Pediatric Diabetes*.

[23] Cox C., Alyahyawi N., Ornstein A., Cummings E. A. (2021). Experience of caring for a child with type 1 diabetes mellitus in a food-insecure household: a qualitative evaluation. *Canadian Journal of Diabetes*.

[24] Inman M., Daneman D., Curtis J. (2016). Social determinants of health are associated with modifiable risk factors for cardiovascular disease and vascular function in pediatric type 1 diabetes. *The Journal of Pediatrics*.

[25] Malik F. S., Liese A. D., Reboussin B. A. (2023). Prevalence and predictors of household food insecurity and supplemental nutrition assistance program use in youth and young adults with diabetes: the SEARCH for diabetes in youth study. *Diabetes Care*.

[26] Morone J. F., Cronholm P. F., Teitelman A. M., Hawkes C. P., Lipman T. H. (2022). Underrepresented voices: impacts of social determinants of health on type 1 diabetes family management in single-parent, black families. *Canadian Journal of Diabetes*.

[27] Smaldone A., Ritholz M. D. (2011). Perceptions of parenting children with type 1 diabetes diagnosed in early childhood. *Journal of Pediatric Health Care*.

[28] Malik F. S., Yi-Frazier J. P., Taplin C. E. (2018). Improving the care of youth with type 1 diabetes with a novel medical-legal community intervention: the diabetes community care ambassador program. *The Diabetes Educator*.

[29] Chang W., Oo M., Rojas A., Damian A. J. (2021). Patients’ perspectives on the feasibility, acceptability, and impact of a community health worker program: a qualitative study. *Health Equity*.

[30] Davoust M., Drainoni M.-L., Baughman A. (2021). “He gave me spirit and hope”: client experiences with the implementation of community health worker programs in HIV care. *AIDS Patient Care and STDs*.

[31] Lipman T. H., Hawkes C. P. (2021). Racial and socioeconomic disparities in pediatric type 1 diabetes: time for a paradigm shift in approach. *Diabetes Care*.

[32] Bonevski B., Randell M., Paul C. (2014). Reaching the hard-to-reach: a systematic review of strategies for improving health and medical research with socially disadvantaged groups. *BMC Medical Research Methodology*.

[33] Abrahamse M. E., Niec L. N., Junger M., Boer F., Lindauer R. J. L. (2016). Risk factors for attrition from an evidence-based parenting program: findings from the Netherlands. *Children and Youth Services Review*.

[34] O’Brien R. A., Moritz P., Luckey D. W., McClatchey M. W., Ingoldsby E. M., Olds D. L. (2012). Mixed methods analysis of participant attrition in the nurse-family partnership. *Prevention Science*.

