# Supplementary material for: Adverse Social Determinants of Health in Children with Newly Diagnosed Type 1 Diabetes: A Potential Role for Community Health Workers
Source: Pediatr Diabetes. 2024 Jan 23;2024:8810609. doi: 10.1155/2024/8810609 (PMC12017050; doi:10.1155/2024/8810609)
Supplement: Supplementary Materials — Semistructured interview guide. [file 8810609.f1.docx]

Supplementary Appendix

Semi-Structured Interview Guide

| Topic | Questions | Prompts |
| --- | --- | --- |
| Introduction and Warmup | I’d like to start by learning about you and your family.  Tell me about yourself, who you are, what’s important to you.  What kinds of things do you do every day?  Tell me about your family. |  |
| How are they doing with diabetes care?  Trying to get an impression of how they feel about their ability to manage diabetes | Can you tell me how you and your family were affected by (the subject’s name)’s diagnosis of diabetes? How has this changed or not changed in the last few months? |  |
|  | Different families have different parts of diabetes care that they find more difficult or easier. Can you tell me about some of the challenges you have faced since going home from the hospital? | - How was integrating back into school? |
|  | What people, groups, or other resources have you reached out to in order to get help with these issues? | - Was this helpful? - Did they reach out to Diabetes Center? If not, why? |
| Examining social determinants of health | What other “things,” in general, are going on in your life and your family’s life that make it easier or harder to take care of (subject’s name) ’s diabetes? | - If they don’t know what to say: can prompt with work, time, money, stress, support systems |
|  | I noticed you indicated you needed help with (SDOH need indicated on HealthLeads USA SDOH screen)  What people, groups, or other resources have you reached out to in order to get help with these issues? | - Did CHW help with these? - Whatever they reached out to- was it helpful? |
| Describing the CHW relationship and program  (Intervention group only) | How would you describe what _____ (name of CHW) does to someone who hasn’t heard of a CHW before?  I’d like to learn about your experience working with (name of CHW). Where, when you meet, what you talk about, what you do?  How do you think working with a CHW has affected you and your family’s ability to take care of ____ (subject’s name)?  How could we improve the CHW program? |  |
| Conclusion | Is there anything I didn’t ask you about that you would like to share today? | |
